# Deforestation, agriculture and farm jobs: a good recipe for *Plasmodium vivax* in French Guiana

**DOI:** 10.1186/1475-2875-12-90

**Published:** 2013-03-11

**Authors:** Célia Basurko, Christophe Demattei, René Han-Sze, Claire Grenier, Michel Joubert, Mathieu Nacher, Bernard Carme

**Affiliations:** 1 Centre d’Investigation Clinique Epidémiologie Clinique Antilles Guyane CIC-EC CIE 802, Centre Hospitalier de Cayenne, rue des Flamboyants, BP 6006, Cayenne, F- 97306, French Guiana; 2 EA3593, UFR Médecine - Université des Antilles et de la Guyane, Cayenne, French Guiana; 3 Biostatistiques, épidémiologie, santé publique et information médicale (BESPIM), CHU de Nîmes, Nîmes, France; 4 Département des centres de santé, Cayenne General Hospital, Cayenne, French Guiana; 5 Laboratoire Hospitalo-Universitaire de Parasitologie et Mycologie Médicale, Cayenne General Hospital, Cayenne, French Guiana

**Keywords:** Malaria, *Plasmodium vivax*, Spatial/temporal clustering, Agriculture, Deforestation, French Guiana

## Abstract

**Background:**

In a malaria-endemic area the distribution of patients is neither constant in time nor homogeneous in space. The WHO recommends the stratification of malaria risk on a fine geographical scale. In the village of Cacao in French Guiana, the study of the spatial and temporal distribution of malaria cases, during an epidemic, allowed a better understanding of the environmental factors promoting malaria transmission.

**Methods:**

A dynamic cohort of 839 persons living in 176 households (only people residing permanently in the village) was constituted between January1st, 2002 and December 31st, 2007.

The information about the number of inhabitants per household, the number of confirmed cases of *Plasmodium vivax* and house GPS coordinates were collected to search for spatial or temporal clustering using Kurlldorff’s statistical method.

**Results:**

Of the 839 persons living permanently in the village of Cacao, 359 persons presented at least one vivax malaria episode between 2002 and 2007. Five temporal clusters and four spatial clusters were identified during the study period. In all temporal clusters, April was included. Two spatial clusters were localized at the north of the village near the Comté River and two others localized close to orchards.

**Conclusion:**

The spatial heterogeneity of malaria in the village may have been influenced by environmental disturbances due to local agricultural policies: deforestation, cultures of fresh produce, or drainage of water for agriculture. This study allowed generating behavioural, entomological, or environmental hypotheses that could be useful to improve prevention campaigns.

## Background

In a malaria endemic area, the repartition of patients is neither constant in time nor homogeneous in space [1]. Identifying the determinants that explain spatial and temporal heterogeneity may allow a better understanding of the environment that is favourable for the transmission of *Plasmodium* to humans, particularly in areas where transmission is irregular, or during malaria epidemics. What is true at the scale of a country or a county may also be true at the scale of a village [2,3].

The ecological characteristics of the vectors of malaria in French Guiana have not been completely elucidated [4]. Over a period of 14 years there were no cases of malaria in the village of Cacao in French Guiana. Then, between 2002 and 2007, an epidemic caused essentially by *Plasmodium vivax* occurred, affecting almost 43% of the inhabitants of the village [5].

The spatial representation of patients in the village of Cacao in French Guiana during this epidemic raised several possible scenarios on the environmental factors promoting the encounter between humans and malaria parasites.

## Methods

### Study zone and population

The study zone has a humid, tropical environment and is covered by a dense forest with large amounts of aerial biomass. Humidity is about 90% all year round. The village of Cacao is situated near the coast of French Guiana, 80 km east of Cayenne. The mountaintops are low, at an altitude ranging from 30 to 400 m.

**Figure 1:**
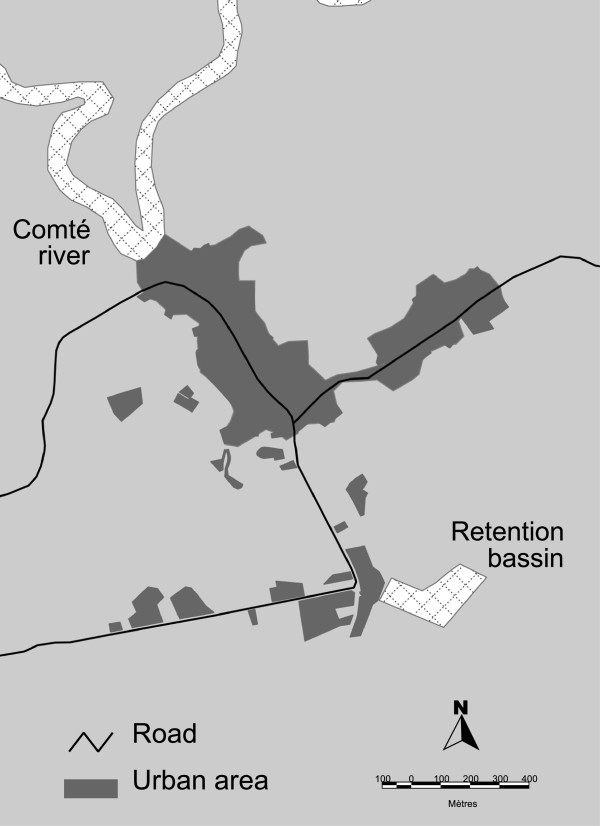
Map of Cacao village.

A dynamic cohort was constituted between January 1st, 2002 and December 31st, 2007. The study included 839 persons living in 176 households. Only patients residing permanently in the village for the duration of the study were included for analysis. The average number of persons living under the same roof was 5 (+/- 3) with a maximum of 12. The Cacao population remained stable during the time of the study. The village of Cacao has a principal road that leads to Cayenne and ends near the Comté River. At the south-east lie water retention basins for irrigation and at the north lies the Comté River (Figure 1).

The inhabitants were followed up by the medical team of the health center. There was only one health facility in the village where screening for malaria could be performed.

Malaria was defined as fever (temperature ≥38°C at the time of consultation or during the pervious 48 hours) associated with a positive thin and/or thick blood smear for *Plasmodium* asexual forms. The parasitological results were validated by a biologist from the University Laboratory of Parasitology and Mycology in Cayenne General Hospital. None of the villagers received malaria prophylaxis. For each household, the information about number of inhabitants, number of malaria cases and GPS coordinates, were collected. The study used data from a department of Cayenne General Hospital declared to the “Commission Nationale de l’information et des libertés” (CNIL authorization no. 1445406).

### Cluster analysis

A cluster can be defined as a collection of unusual events in time or space within a specific population. The search for spatial or temporal clustering was performed using Kurlldorff’s statistical method [6] of temporal/spatial scan under the hypothesis that cases follow a Poisson distribution in time and space.

For temporal clusters, the analysed data are the time of the occurrence of the malaria attacks (since the beginning of the study). For spatial clusters, it is the frequency of malaria in every home during the study period. Scan statistics were used to detect the clusters of malaria cases (purely temporal or purely spatial) by gradually scanning a window across time (interval of time) or space (circular window). The most likely cluster with the maximum likelihood was the cluster least likely to be due to chance. High-risk cluster detection was performed by comparing the observed number of cases inside the window with the expected number at each location.

The purely spatial scan statistic imposed a circular window on the map of the village. The window was in turn centered on each of several possible grid points positioned throughout the study region. For each point, the radius of the window varied continuously in size from zero to some upper limit (50%). This circular window was flexible both in its location and size, while each was a possible candidate cluster. The temporal scan statistic used a window that moved in the dimension of time, defined in the same way as the space scan statistic. This time window was flexible in both start and end date. For each window, the method tests the null hypothesis against the alternative hypothesis that there is an elevated risk of malaria cases within, compared with outside, the window.

A malaria case corresponded to a patient having had at least one case of vivax malaria. The date of the first positive sample for the search of *P. vivax* was retained as the case date. The home of the case was considered as the place of infection by an infected *Anopheles*. The search was performed for the entire duration of the study (N=839) on the population at risk of the considered year, which corresponds to the total initial population diminished by the cases of previous years. The objective of analysis per year is the study of malaria transmission dynamic in this Amazon region.

Tests of statistical significance of the identified clusters were based on likelihood ratio tests, with P-values obtained by 9999 Monte Carlo replications. Statistical significance was set at p<0.05. Analyses were performed with R 2.9.2 and SaTScan™ software v8.0.

## Results

Of the 839 persons living permanently in the village of Cacao, 359 persons presented at least one vivax malaria episode between 2002 and 2007. Forty-seven percent presented at least one malaria relapse during the study (one relapse for 101 persons, two for 21 and three for 2). Half of the persons were more than 21.5 years of age and the sex ratio (M/F) was 1.3. During the same period, the number of persons residing temporarily in the village having also had at least one malaria episode was 267. There were mostly men (sex ratio = 5.2) of more than 21 years of age (75%).

**Figure 2:**
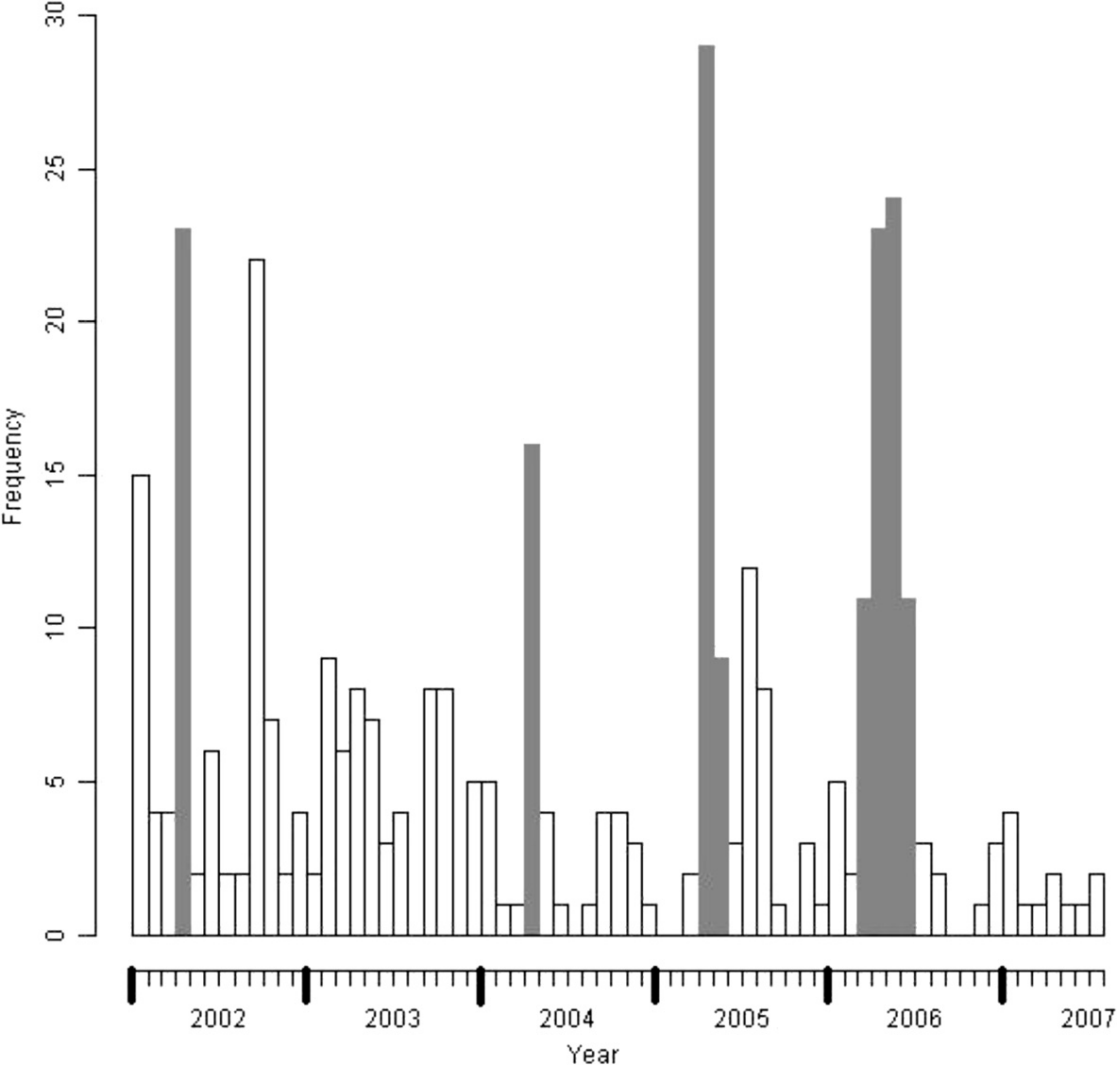
**Number of monthly cases of *P. vivax *malaria between 2002 and 2007 and representation of temporal clusters (in gray on the histogram) per year detected during the vivax epidemic study period.**

During the study period, 44 patients on the 359 Cacao inhabitants included in the cohort had a malaria episode between April 17th and May 26th, 2006 (significant temporal cluster p=0.001; relative risk = 7.51). Temporal clusters that were significant in 2002, 2004, 2005, and 2006 are detailed in Table 1 and represented in Figure 2. There were no clusters in 2003 and 2007.

**Table 1:** **Description of temporal clusters by year**, **detected between 2002 and 2007 during the *vivax *malaria epidemic**

**Study period**	**Number of cases / population at risk**	**Time cluster**			
		**Time frame**	**Number of cases**	**Relative risk**	**P-value**
2002	93 / 839	22/4–30/4	19	10.16	0.001
2004	41 / 686	6/4–28/4	16	9.54	0.001
2005	68 / 645	4/4–16/5	37	8.94	0.001
2006	85 / 577	19/3–27/6	67	9.73	0.001

**Figure 3:**
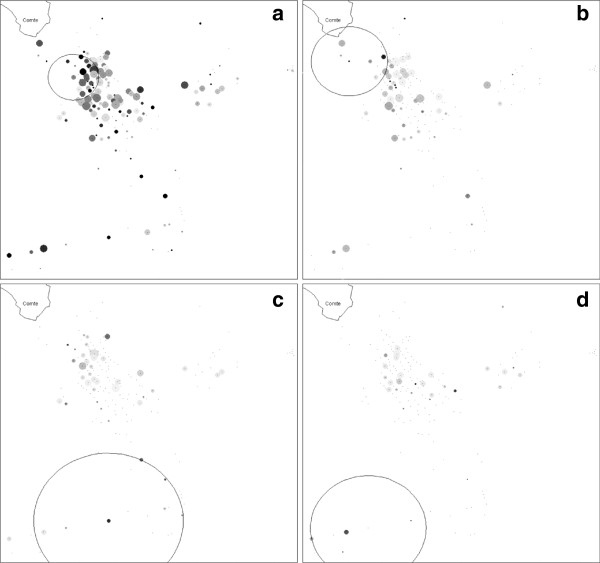
**Detection of spatial clusters between 2002 and 2007 (a) in 2002 (b) in 2003 (c) and in 2004 (d) in the Cacao are during the *P. vivax *malaria epidemic.** Each building is represented by a circle. The disk size is proportional to the number of inhabitants in the building and the level of grey is proportional to the density of cases in the building. Significant clusters are indicated with a circle.

The graphs of Figure 3 represent the spatial repartition of cases. Each building is represented by a point. A Black disk represents building where all occupants were infected and a point building where nobody was infected. Significant clusters are indicated with a circle.

During the study period (1 January, 2002 to 31 December, 2007) the 359 patients having presented at least one malaria episode, showed a tendency to spatial aggregation (Figure 3a) at the north of the village near the Comté River, in an area with a radius of 129 m, concentrating 93 cases among 151 persons in 27 houses (p=0.079). Table 2 synthesizes the results of spatial clusters that were only observed during the first three years of the study (2002 to 2004). Indeed, in 2005, 2006, and 2007 no significant spatial cluster was detected.

**Table 2:** **Description of spatial clusters per year detected between 2002 and 2007 during the *P.vivax *malaria epidemic**

**Period**	**Number of cases / population at risk**	**In the spatial cluster**				
		**Number of inhabitants**	**Number of cases**	**Radius (in m)**	**Number of houses**	**P**
2002	93 / 839	42	17	194	10	0,003
2003	60 / 746	45	13	380	14	0,033
2004	41 / 686	18	8	294	6	0,005

## Discussion

Kulldorff’s spatial analysis allowed the detection of four circular spatial clusters. By using detection methods without predetermined shapes, the detected clusters would probably have had shapes more adapted to the spatial structure of data [6] by avoiding the inclusion of statistical units close to the limits of the studied spatial domain but without risk (edge effects). However, Kulldorff’s statistic relies on the likelihood ratio and has the advantage of avoiding multiple testing when compared with other screening methods.

This study is based on malaria case data in order to find conditions favourable to the transmission of the parasite to humans [2,3,7-9]. It is an indirect method that can be interesting in the cases where longitudinal entomological data are not available or when entomological investigations were non-contributive.

The four clusters detected were correlated with different factors that enhance transmission at two different areas of the village. One was situated at the north-east of the village, near market gardening cultures, near the Comté River. Drainage ditches delimitating the agricultural properties were constantly filled with rainwater, irrigation water, or water from the Comté’s flooding. These collections of water in opened-up areas of rainforest, motioned by a weak current, could constitute excellent breeding sites for anopheline larvae, in particular *Anopheles darlingi* in the Amazon region [10,11]. The other geographic area was localized at the south-west of the village where the building density was weak. This second area was close to fruit cultures and burnt forest, a technique for reducing the forest cover in order to increase farming surfaces.

The anthropization of natural environments radically changes biotopes and may lead to a reduction of the distance between man and parasite (sylvatic vector emergence, disappearance of animal hosts, creation of new breeding sites) [12,13]. The occurrence of a malaria epidemic in Cacao could have been influenced by environmental disturbances due to local agricultural policies [14,15]. Indeed the transition from tropical forest to agricultural land following massive deforestation [15-17] can remodel ecological niches. Uncontrolled deforestation, irrigation, or drainage of water for agriculture could have increased vector breeding sites but could also have influenced human behaviour: agriculture has attracted several seasonal workers from the east of French Guiana. This hypothesis is supported by the observation of a high proportion of young men in non-residents (from the East of French Guiana) in the malaria cohort at the Cacao health centre (sex ratio = 5.2). The building of the road connecting Brazil and Cacao may have also facilitated cross-border movements of people for economic reasons and the arrival of hosts carrying the parasite from malaria-endemic areas [18-20]. Malaria diagnosed by the Health Centre of Cacao in non-resident persons corresponded to “border malaria” (young adult males) [20,21].

Taking the short incubation period into account for *P. vivax* new infections and relapses observed in French Guiana (relapse pattern of *P. vivax* Chesson (tropical) strain) [22,23], the transmission period of the parasite occurred according to the temporal clusters during the rainy season [3,24,25]. The period also corresponds to the rambutan fruit-picking period, to logging and deforestation activities often carried out by seasonal workers.

Spatial heterogeneity of malaria in the village can be explained by different risk factors. The absence of use of mosquito nets, or repellents, the existence of outside kitchens, late bedtime or early rising habits (agricultural work in the fields) can theoretically influence the risk of *Anopheles* bites.

## Conclusion

The study of the repartition of malaria cases in a determined geographical zone allowed the identification of locations where clustering of cases was unlikely to be due to chance [6,8]. Behavioural, entomological or environmental hypotheses were generated that could be useful on two levels: on the field by improving prevention campaigns (vector control programmes) and in research for the understanding of the ecology of the *Anopheles* mosquitoes in the region.

## Competing interests

The authors declare that they have no competing interests.

## Authors’ contributions

CB participated in the research design, performed data analysis and interpretation, and prepared the manuscript. CD participated in the statistical analysis and manuscript. RHS, CG, MJ participated in interpretation of data and manuscript as well. MN provided guidance on data analyses and was involved in the interpretation of data and manuscript revision. BC was responsible for data collection, initiated the study, and was involved in the interpretation of data and manuscript revision. All authors read and approved the final manuscript.
